# Periodontal Disease Elevates IL-6 Levels During Initial Symptoms of COVID-19

**DOI:** 10.3390/diagnostics15131650

**Published:** 2025-06-28

**Authors:** Ruth Rodríguez-Montaño, Tonatiuh Abimael Baltazar-Díaz, Oscar Hernández-Mora, Mario Alberto Isiordia-Espinoza, Fatima Del Muro-Casas, Rogelio González-González, Ronell Bologna-Molina, Sandra López-Verdín

**Affiliations:** 1 Departamento de Salud-Enfermedad como Proceso Individual y Colectivo, del Centro Universitario de Tlajomulco, de la Universidad de Guadalajara (CUTLAJO-UdeG), Tlajomulco de Zuñiga 45641, Jalisco, Mexico; ruth.rodriguez@academicos.udg.mx; 2 Instituto de Investigación en Odontología, Departamento de Clínicas Odontológicas Integrales, Universidad de Guadalajara, Guadalajara 44100, Jalisco, Mexico; 3 Instituto de Investigación en Enfermedades Crónico-Degenerativas, Departamento de Biología Molecular y Genómica, Centro Universitario de Ciencias de la Salud, Universidad de Guadalajara, Guadalajara 44100, Jalisco, Mexico; tonatiuh.baltazar@academicos.udg.mx; 4 Especialidad de Periodoncia, Departamento de Clínicas Odontológicas Integrales, Universidad de Guadalajara, Guadalajara 44100, Jalisco, Mexico; leobardo.hernandezm@alumnos.udg.mx; 5 Departamento de Clínicas, División de Ciencias Biomédicas, Centro Universitario de los Altos, Universidad de Guadalajara, Tepatitlán de Morelos 47620, Jalisco, Mexico; mario.isiordia@academicos.udg.mx; 6 Dental Academic Unit, Research Department, Universidad Autónoma de Zacatecas, Ciudad de Zacatecas 98160, Zacatecas, Mexico; fatima.delmurocasas@uaz.edu.mx; 7 Department of Research, School of Dentistry, Universidad Juárez del Estado de Durango, Durango 34000, Durango, Mexico; rogelio.gonzalez@ujed.mx; 8 Molecular Pathology Area, School of Dentistry, Universidad de la República, Montevideo 11600, Uruguay

**Keywords:** interleukin-6, periodontitis, COVID-19, saliva, inflammation

## Abstract

**Background:** Research suggests that periodontal disease may exacerbate symptoms of coronavirus disease (COVID-19). The etiology of this condition has been associated with cytokines such as IL-6. The inflammatory response in COVID-19 can be partially attributed to periodontopathic bacteria and their metabolites. Furthermore, the aspiration of periodontal pathogens and the stimulation of ACE2 expression may lead to an increased production of inflammatory cytokines, potentially worsening COVID-19 symptoms in patients with periodontitis. **Materials and Methods:** A cross-sectional study was conducted involving patients with both periodontal disease and COVID-19, patients with either condition alone, and healthy subjects. All participants underwent RT-PCR testing for SARS-CoV-2, and a self-reported periodontal disease (Self-RPD) questionnaire was administered. Saliva samples were collected to assess IL-6 levels using the ELISA technique. **Results:** A total of 28 patients were classified as COVID-19/Self-RPD+, 32 patients had only COVID-19, 25 were Self-RPD+ only, and 17 were healthy controls. The COVID-19/Self-RPD+ group frequently exhibited symptoms such as fever, body aches, nasal congestion, and olfactory disturbances and showed significantly higher IL-6 levels compared to the other groups. Cough with phlegm was significantly more frequent in the COVID-19-only group. Additionally, IL-6 levels in saliva were elevated in patients with nasal congestion and in those with 11 or more symptoms in the Self-RPD+ group.

## 1. Introduction

In late December 2019, the World Health Organization (WHO) announced the identification of a novel coronavirus (CoV), provisionally designated as severe acute respiratory syndrome coronavirus 2 (SARS-CoV-2), in Wuhan, a major city in central China. This virus subsequently spread worldwide. The WHO declared SARS-CoV-2 a Public Health Emergency of International Concern on 30 January 2020, and later classified it as a pandemic in March 2020 [1], due to its association with a spectrum of human respiratory tract infections ranging from mild colds to severe, potentially fatal respiratory distress syndrome (COVID-19) [2].

The virus primarily spreads through coughing, sneezing, inhalation of respiratory droplets, and contact with the mucous membranes of the mouth, nose, and eyes, making it highly contagious. Human-to-human transmission occurs via various common routes, including direct contact, airborne transmission through aerosols, and exposure during medical procedures. These factors, especially in dentistry, posed significant challenges at the pandemic’s onset before vaccines were available, owing to the potential for virus transmission during dental procedures and within dental settings. This led to the mandatory or voluntary suspension of routine dental care [3] in both private practices and institutional facilities.

Initial hypotheses regarding the relationship between periodontitis and COVID-19 were based on the theoretical framework of characteristic inflammation in periodontitis, driven by microbial agents, proteinases, cytokines, and host factors such as comorbidities, resulting in the loss of periodontal attachment [4,5]. Due to the inability to directly contact patients during the height of the pandemic, original research was largely based on database analyses or unvalidated questionnaires, which began to corroborate these hypotheses [6,7].

The possible role of the oral cavity as an entry point for the virus emerged because the oral mucosa was identified as having a high expression of angiotensin-converting enzyme II (ACE2) [8], a key receptor in viral pathogenesis alongside the spike protein subunit 1 and priming by transmembrane protease serine-2 [9]. SARS-CoV-2 infects target cells by binding its spike (S) glycoprotein to the ACE2 receptor, leading to the downregulation of ACE2. This disrupts the enzymatic activity of ACE2, which is essential for protecting against organ injury by cleaving and degrading angiotensin II (Ang II) into Ang 1–7. The resulting accumulation of Ang II activates the angiotensin II type 1 receptor (AT1R), promoting leukocyte recruitment and increasing proinflammatory cytokine production [10]. Conversely, ACE2 expression in oral epithelial cells helps regulate cytokine levels by downregulating proinflammatory cytokines such as IL-6, IL-7, IL-2, MCP-1, TNF-α, and IL-1β [8].

The inflammatory processes induced by both COVID-19 and periodontitis involve various proinflammatory cytokines, including IL-1, INF-γ, IL-17, IL-8, and IL-6 [5]. Recent studies have demonstrated a significant association between elevated IL-6 levels and severe COVID-19 outcomes, indicating that higher IL-6 concentrations correlate closely with increased disease severity and complications [11,12,13]. In periodontitis, the inhibition of IL-6 has been shown to reduce inflammatory bone loss in experimental models [14]. Likewise, patients with periodontitis exhibit elevated IL-6 levels, which significantly decrease following non-surgical treatment [15].

Saliva is an easily obtainable body fluid through noninvasive techniques and holds potential for diagnostic applications. Whole saliva comprises secretions from the salivary glands as well as gingival crevicular fluid derived from the epithelial mucosa [16]. SARS-CoV-2 has been detected in minor salivary glands, including ducts and acini, which act as sites for viral replication and reservoirs, facilitating viral persistence and potential dissemination within the body and to others. Furthermore, viral load in saliva correlates with the presence of symptoms [17]. Previous research has also documented ACE2 receptor expression in human gingival fibroblasts [18]. Moreover, the disruption of the oral microbiome may be influenced by factors such as pregnancy, which has been associated with an increased presence of bacterial species linked to gingival diseases, including *Fusobacterium nucleatum*, *Prevotella intermedia*, *Porphyromonas gingivalis*, and *Aggregatibacter actinomycetemcomitans*. This positions pregnancy as a potential risk factor for SARS-CoV-2 infection. Nevertheless, a prospective cohort study suggests that SARS-CoV-2 infection during the first 10 days of embryonic implantation does not pose a risk of early pregnancy complications [19,20].

A cytokine storm has been associated with COVID-19 severity; however, research exploring the relationship between IL-6 levels, periodontitis, and COVID-19 has been largely limited to hospitalized patients [21,22,23], neglecting important epidemiological studies necessary for understanding the etiology and associations of different disease conditions and stages. In this context, self-reported periodontal disease has been considered an efficient surveillance tool for studying ambulatory populations [24].

This study aims to establish the association of salivary IL-6 levels with COVID-19 and periodontal disease status in unvaccinated patients who underwent COVID-19 testing during the acute phase of the pandemic in 2020 and 2021.

## 2. Materials and Methods

### 2.1. Study Design and Ethical Considerations

A cross-sectional study was conducted between December 2020 and January 2021 involving individuals aged 20 to 75 years who met the criteria for a COVID-19 diagnosis. The study adhered to ethical and biosafety standards outlined in the Declaration of Helsinki and was approved by the Ethics Committee of the Center for Multidisciplinary Research in Health at the University of Guadalajara (CEI-2020-10). Written informed consent was obtained from all participants.

### 2.2. Participant Recruitment and Data Collection

Participants were recruited at the COVID-19 Diagnostic Center of the University Center-Tonalá, University of Guadalajara. Due to time constraints imposed by pandemic-related protocols and the requirements of the institutional ethics committee, a non-probability convenience sampling method was used. A total of 102 participants were recruited during the authorized data collection period. Structured questionnaires were administered via a call center to collect detailed demographic and epidemiological data, including age, sex, and place of residence within the preceding 15 days.

#### Eligibility for RT–qPCR Testing for SARS-CoV-2

Study inclusion criteria required participants to have reported symptoms of mild illness within the previous 15 days, as defined by the Centers for Disease Control and Prevention (CDC) [25]. These symptoms included fever, cough, shortness of breath or difficulty breathing, sore throat, nasal congestion or runny nose, new loss of taste or smell, fatigue, muscle or body aches, headache, nausea or vomiting, and diarrhea.

Exclusion criteria comprised participants under 18 years old, pregnancy, use of anticoagulant medications, blood disorders (e.g., hemophilia), and autoimmune diseases. The same criteria applied uniformly to all groups.

### 2.3. Sample Collection Procedure

In accordance with public health protocols to prevent and control COVID-19, three stations were established at a designated university site. Station One involved patient registration, an invitation for them to participate, and obtaining their informed consent. Station Two involved sample collection using intranasal swabs for COVID-19 diagnosis. At Station Three, participants deposited their signed consent forms in a collection box. Those who consented received a kit containing two microtubes for saliva collection and a questionnaire on self-reported periodontal disease.

### 2.4. Self-Reported Periodontal Disease

Participants were blinded to their COVID-19 test results at the time of data collection. Periodontal health was assessed using the Self-Reported Periodontal Disease (Self-RPD) questionnaire validated by Khader et al. The questionnaire consists of six items:

Q1: Do you have periodontal or gum disease?
Q2: Have you ever been told by a dentist that you have periodontal/gum disease with bone loss?
Q3: Do you notice any areas that appear redder than usual?
Q4: Do you experience tooth mobility?
Q5: Do you have food impaction between your teeth?
Q6: Do you notice that your teeth appear to be getting longer?

Participants scoring ≥ 2 were classified as having self-reported periodontal disease [26].

### 2.5. Saliva Collection and IL-6 Quantification

Saliva samples were collected on the same day as the nasopharyngeal swab in sterile containers and placed in plastic bags after disinfection with 70% ethanol. Saliva samples were treated with 4 µL of protease inhibitor to prevent degradation of the cytokine of interest. Then, they were centrifuged for 10 min at 10,000 rpm to remove cell and food debris and stored at −80 °C until analysis in the year 2023, two years after their collection. IL-6 concentrations were quantified using the LEGEND MAX™ Human IL-6 ELISA Kit (Biolegend Way, San Diego, CA, USA, cat. no. 430507).

All reagents were brought to room temperature before use. Samples and standards were analyzed in duplicate or triplicate, and a standard curve was prepared by serial 1:2 dilutions from 500 pg/mL to 7.8 pg/mL using Assay Buffer A (Biolegend Way, San Diego, CA, USA) as diluent and zero standard. The microplate was washed four times with 1X Wash Buffer (Biolegend Way, San Diego, CA, USA) and dried on absorbent paper. Then, 50 µL of Assay Buffer A and 50 µL of either standards or samples were added to each well. The plate was incubated for 2 h at room temperature with shaking (200 rpm), followed by washing. Next, 100 µL of detection antibody was added and incubated for 1 h, followed by another wash. Subsequently, 100 µL of Avidin-HRP solution was added and incubated for 30 min. After five additional washes with 30 s soaking, 100 µL of substrate solution was added, and it was incubated in the dark for 15 min. The reaction was stopped with 100 µL of stop solution, and absorbance was measured at 450 nm (with correction at 570 nm if available).

### 2.6. Sample Collection and RT–qPCR Detection of SARS-CoV-2

Two nasopharyngeal swab specimens were collected by trained personnel. A sterile nasopharyngeal swab was introduced in one nostril and then withdrawn. Another sterile swab was introduced in the mouth until reaching the oropharynx, rubbed on the posterior wall for 10–15 s and withdrawn, according to published guidelines [27]. Both swabs were placed into 3 mL of viral transport media and processed for SARS-CoV-2 RNA determination. SARS-CoV-2 RNA for RT-PCR from swabs was extracted with the PureLink Viral RNA/DNA Mini Kit (Invitrogen, Waltham, MA, USA, cat no. 12280050), according to manufacturer’s indications. RT-PCR assay was performed with DeCoV19 Kit Triplex (Genes2life, Irapuato, Gto, Mexico), which is designed to detect three regions of the virus nucleocapsid gene (N1, N2, and N3) based on the CDC’s recommendations, using a QuantStudio 5 Real-Time PCR System (ThermoFisher, Waltham, MA, USA, cat. no. A28573). Blank reactions and human RNase P gene were used as quality-control RNA samples and included in all reactions. The PCR results were obtained within 24 h.

### 2.7. Statistical Analysis

Statistical analyses were conducted using SPSS version 25. The Shapiro–Wilk test assessed the normality of numerical variables. Categorical variables were analyzed using the chi-square (χ^2^) test, while continuous variables were analyzed with the Mann–Whitney U test. A *p*-value < 0.05 was considered statistically significant, with a 95% confidence interval.

## 3. Results

In the present study, salivary levels of interleukin-6 (IL-6) were evaluated in patients who attended the COVID-19 Diagnostic Center at the University of Guadalajara and completed a self-reported periodontal disease (Self-RPD) questionnaire. The study population consisted of 102 participants, with a higher proportion of females (56 participants; 54.9%) compared to males (46 participants; 45.1%). The mean age was 38.1 ± 15.1 years.

All 102 participants consented to take part in the study. Among them, 60 individuals (58.8%) tested positive for COVID-19, while 42 (41.2%) tested negative. Based on their COVID-19 status and Self-RPD results, participants were classified into four groups: (1) COVID-19-positive and Self-RPD-positive (COVID-19/Self-RPD+), (2) COVID-19-positive only (COVID-19), (3) Self-RPD-positive only (Self-RPD+), and (4) healthy subjects (HSs).

Comorbidities were reported by 22 participants (21.5%), with 13 individuals (12.7%) reporting diabetes and 9 (8.8%) reporting hypertension.

The Self-RPD questionnaire was successfully used to evaluate suggestive changes related to periodontal disease. A total of 53 participants were classified as Self-RPD-positive. Among them, 28 individuals (46.7%) also tested positive for COVID-19, and 25 (59.5%) were COVID-19-negative. Importantly, our questionnaire items (Qs1, Qs2, Qs5, and Qs6) showed a significantly higher frequency of positive responses in group 1 (COVID-19/Self-RPD+) and group 3 (Self-RPD+) (*p*-values = 0.000, 0.001, 0.000, and 0.000, respectively), as shown in Table 1.

### 3.1. Clinical Characteristics and COVID-19 Symptomatology Among Groups

Notably, several of the clinical characteristics and symptoms evaluated exhibited statistically significant differences, underscoring the relevance of the findings. Fever (18 participants, 64.3%), body aches (11, 39.3%), olfactory disturbances (17, 60.7%), and nasal congestion were significantly more frequent in the COVID-19/Self-RPD+ group. In contrast, productive cough (cough with phlegm) was more prevalent in participants with COVID-19 only (Table 2).

### 3.2. Salivary IL-6 Levels According to COVID-19 and Self-RPD Status Groups

**Figure 1:**
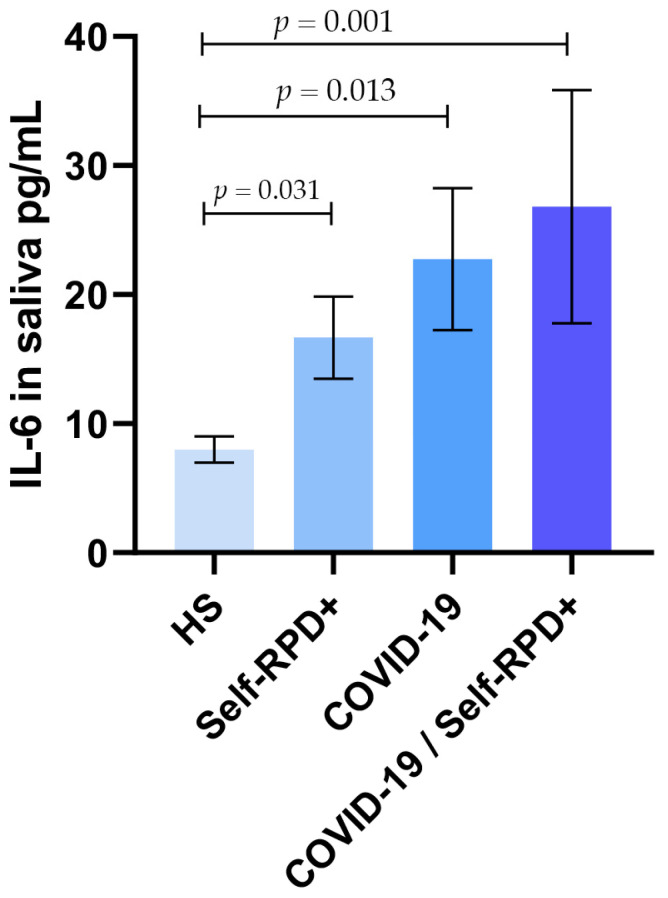
Data are presented as mean ± standard deviation of salivary IL-6 levels. A Mann–Whitney U test was performed, and a *p*-value ≤ 0.050 was considered statistically significant.

We found, as expected, that IL-6 levels in the COVID-19/Self-RPD+ group (26.8 pg/mL ± 43.4 pg/mL) were significantly higher than those in the COVID-19 only and Self-RPD+ groups (22.7 pg/mL ± 29.1 pg/mL and 16.6 pg/mL ± 14.9 pg/mL, respectively). Furthermore, as shown in Figure 1, IL-6 levels were significantly lower in the healthy subject (HS) group (7.9 pg/mL ± 3.4 pg/mL) compared to subjects with either Self-RPD+ or COVID-19 alone, as well as those presenting both conditions.

### 3.3. Salivary IL-6 Levels According to Symptoms

Considering previous results, we decided to stratify the IL-6 results according to the evaluated symptoms. Taking into account the analyzed variables, only patients with nasal congestion showed significantly higher IL-6 levels (28.2 pg/mL ± 39.0 pg/mL vs. 12.0 pg/mL ± 8.1 pg/mL; *p* = 0.013). Similarly, when analyzed within each group, this symptom showed a trend toward elevated IL-6 levels in the Self-RPD+ group (32.9 pg/mL ± 51.1 pg/mL vs. 12.9 pg/mL ± 5.1 pg/mL), although this difference was not statistically significant (*p* = 0.078). Regarding the number of reported symptoms, similar averages were observed across groups. The mean number of symptoms was 7.04 ± 4.61 in the COVID-19/Self-RPD+ group, 4.28 ± 3.02 in the COVID-19 group, 5.95 ± 4.08 in the Self-RPD+ group, and 6.6 ± 3.67 in the healthy subjects.

**Figure 2:**
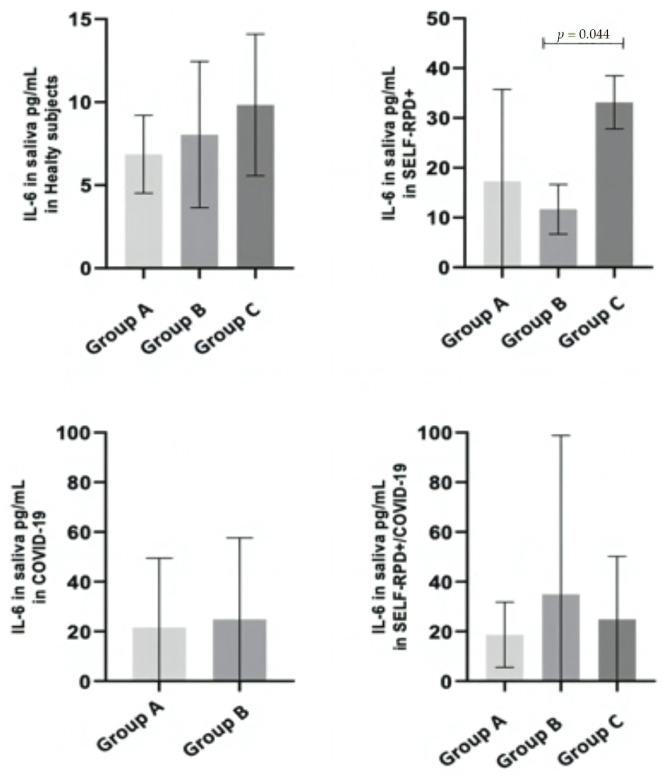
IL-6 levels according to the number of symptoms. Data are presented as mean ± standard deviation of salivary IL-6 levels. A Mann–Whitney U test was performed, and a *p*-value ≤ 0.050 was considered statistically significant. Group A: 1 to 5 symptoms; Group B: 6 to 10 symptoms; Group C: more than 10 symptoms.

Patients in each study group (healthy subject, Self-RPD+, COVID-19, and COVID-19/Self-RPD+ groups) were categorized according to the number of symptoms as follows: Group A, one to five symptoms; Group B, six to ten symptoms; and Group C, eleven or more symptoms. Notably, in the Self-RPD+ group, a significant difference in IL-6 concentration was observed between patients with six to ten symptoms (Group B) and those with more than ten symptoms (Group C) (*p* = 0.044) (Figure 2).

## 4. Discussion

Self-reported questionnaires are a cost- and time-efficient validated tool for assessing the association between periodontal diseases and other systemic conditions [24]. In this study, we observed an inverse relationship in the first two questions concerning patients’ awareness of periodontal disease and whether they had been informed of it by a dentist. Some patients were classified as having periodontal disease despite not having consulted a dentist, as gingivitis is a purely clinical diagnosis. It is therefore unsurprising that individuals may detect gingival changes such as erythema and edema [28].

To date, IL-6 levels have not been evaluated in ambulatory patients concurrently presenting with COVID-19 and periodontal disease, nor has their relationship with the pathognomonic symptoms of COVID-19 been investigated.

The relationship between COVID-19 and periodontitis has been a subject of study, especially during the pandemic period, as periodontal disease can exacerbate COVID-19 symptoms. Consequently, non-surgical periodontal therapies have been evaluated alongside the use of laser treatment, ozone therapy, air polishing, probiotics, and chlorhexidine to reduce bacterial load and the generation of contaminated aerosols, thereby minimizing the risk of transmission and improving clinical outcomes in patients during the pandemic [29]. Periodontal disease is characterized by chronic inflammation and tissue destruction, which can trigger systemic inflammatory responses, potentially affecting the progression of viral infections such as COVID-19. Several studies have explored the relationship between these two conditions, elucidating potential mechanisms through which periodontitis may influence COVID-19 outcomes [30,31,32]. Additionally, shared inflammatory pathways suggest a possible link between periodontitis and COVID-19 complications [33,34,35].

Immune dysregulation in periodontitis, including an increased expression of Galectin-3 and the presence of SARS-CoV-2 in periodontal pockets, may contribute to the severity of COVID-19 in affected individuals [35,36]. It has been reported that patients with moderate to severe periodontitis are more likely to experience COVID-19 complications than those with mild periodontitis [30,37,38,39]. This association is thought to result from shared risk factors between the two diseases, as well as elevated levels of IL-6 and C-reactive protein (CRP) observed in both conditions [40,41,42,43,44,45,46]. Furthermore, a Mendelian randomization study provided evidence supporting a potential causal relationship, indicating that periodontal disease increases both the risk of contracting COVID-19 and the severity of the disease [38].

In the present study, patients with both COVID-19 and Self-RPD exhibited a higher frequency of symptoms such as fever, cough, olfactory disturbance, and nasal congestion, highlighting an increased involvement of the upper airway compared to patients with COVID-19 alone. Additionally, significantly higher IL-6 levels were associated with the concomitant presence of COVID-19 and Self-RPD+, as well as with nasal congestion.

**Figure 3:**
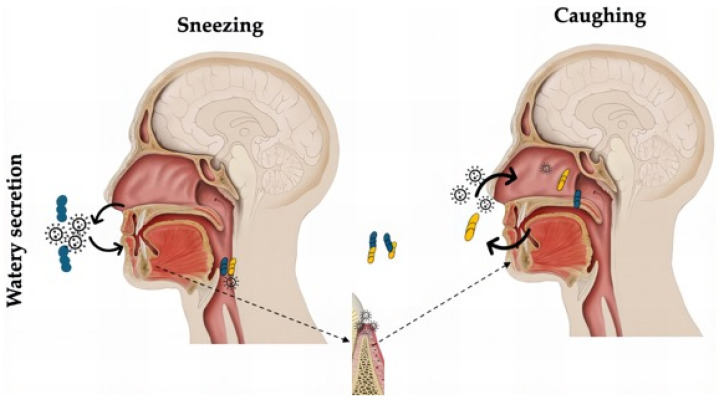
Biological mechanism of feedback in patient with COVID-19 and self-RPD+. When sneezing, water secretions with high load of SARS-CoV2 and soluble IL-6 enter the mouth, which allows for contact between periodontal disease and high levels of IL-6 and cell targets; the virus then passes to lower airways when swallowing saliva. When coughing, the inhalation of saliva droplets with high load of SARS-CoV2 and IL-6 simultaneously supplies the upper airway again.

Based on these findings, we propose a potential biological mechanism involving a cyclical supply of pro-inflammatory mediators to the upper respiratory tract and oral cavity. Patients with COVID-19 and Self-RPD+ may experience sneezing, which leads to the dissemination of contaminated saliva and nasal secretions, promoting an increased SARS-CoV-2 viral load and the accumulation of soluble IL-6 (Figure 3). This hypothesis is further supported by our observation that the healthy subjects presented the lowest IL-6 levels.

Therefore, periodontal disease may amplify the production of inflammatory cytokines in COVID-19 patients, as initial symptoms could be exacerbated by the aspiration of periodontal pathogens and the induction of ACE2 expression. This facilitates SARS-CoV-2 dissemination through ulcerated gingiva, aggravating the cytokine storm and maintaining a state of low-grade chronic systemic inflammation, which may induce pulmonary vascular pathology. Ultimately, this can lead to increased disease severity and a higher risk of mortality in COVID-19 patients [21,23,41,47].

Furthermore, it has been demonstrated that periodontal infections lead to the production of IL-6 and other inflammatory markers in respiratory epithelial cells, suggesting a possible connection between dental health and the consequences of respiratory diseases [48,49]. This is consistent with our results, in which patients with Self-RPD+ exhibiting a high number of symptoms were associated with elevated IL-6 levels, regardless of COVID-19 infection. High levels of IL-6 exacerbate the clinical course of severe COVID-19, as these patients often develop interstitial pneumonia, multi-organ damage, and immune-mediated pulmonary thrombosis. IL-6 has also been linked to COVID-19 severity and mortality, indicating that this cytokine may play a crucial role as an inflammatory mediator in severe cases [12,41,50,51,52].

The overexpression of inflammatory molecules, particularly IL-6 and IL-17, in periodontitis may create an environment conducive to increased COVID-19 susceptibility and severity [23,39,53]. Moreover, the systemic inflammation and immune priming caused by periodontitis could worsen COVID-19 outcomes by exacerbating the immune response to the virus [40].

It is important to note that a potential link between COVID-19 and periodontal disease is supported by shared risk factors such as obesity, age, hypertension, and pregnancy [52]. However, the median age of the population in this study was around thirty or forty, with the exclusion of pregnant women, and there was a low frequency of comorbidities such as diabetes and hypertension. Therefore, the associations observed cannot be attributed to the presence of these risk factors.

IL-6 is a key soluble mediator due to its pleiotropic effects on inflammation, immune response, and hematopoiesis, making it a promising therapeutic target. The investigation of IL-6 inhibitors, such as tocilizumab, as treatment options for COVID-19 patients has been driven by the clinical consequences of elevated IL-6 levels. It has been observed that IL-6 levels following tocilizumab therapy may help distinguish between survivors and non-survivors, suggesting that the pharmacological targeting of IL-6 can reduce hyperinflammatory responses in severe cases. However, the efficacy of IL-6 inhibition remains under investigation, alongside other strategies aimed at enhancing the respiratory immune system, including the use of vitamin D [54,55,56].

To our knowledge, no other study has evaluated the ambulatory population during the pandemic specifically regarding their COVID-19 status and elevated IL-6 levels from the onset of their symptoms. Given the shared potential risk factors between COVID-19 and periodontal disease, further research is needed to fully understand the complex interactions between these two conditions and their implications for patient management.

### Study Limitations

Due to the observational and cross-sectional nature of our study, we are unable to determine whether the elevated IL-6 levels observed in COVID-19/Self-RPD+ patients result from additive inflammatory effects or from a synergistic interaction between the two conditions. We acknowledge that the reliance on self-reported periodontal status through the Self-RPD questionnaire introduces subjectivity and may limit diagnostic accuracy when compared to clinical examinations. The relatively small sample size, along with our inability to establish matched groups, may restrict the generalizability of the findings. Moreover, as participation was voluntary and limited to individuals with access to university-affiliated diagnostic services, there is a risk of selection bias that could influence the representativeness of the sample. These factors should be considered when interpreting the results.

## 5. Conclusions

Upper-airway-related symptoms are commonly observed in patients with both COVID-19 and Self-RPD+, potentially representing the primary mechanism responsible for sustaining elevated IL-6 levels in this group, compared to patients with COVID-19 alone, Self-RPD+ alone, or healthy controls.

Elevated IL-6 levels are known to be associated with systemic inflammation and worse clinical outcomes in COVID-19. The co-occurrence with self-reported periodontal disease suggests a potential compounding inflammatory burden.

Based on these findings, we recommend the implementation of early screening strategies using the Self-RPD tool, particularly in patients with upper airway symptoms, to help identify those at risk of persistent inflammation. This proactive approach could contribute to timely clinical interventions and reduce the burden of systemic complications.

## Figures and Tables

**Table 1:** Test of differences in self-reported periodontal disease among study groups.

		COVID 19/Self-RPD+ (*n* = 28)	COVID-19 (*n* = 32)	Self-RPD+ (*n* = 25)	Healthy Subjects (*n* = 17)	*p*
Qs1	Yes	18 (64.3)	4 (12.5)	23 (92)	0 (0)	**0.000**
	No	10 (35.7)	28 (87.5)	2 (8	17 (100)	
Qs2	Yes	8 (28.6)	0 (0)	6 (24)	0 (0)	**0.001**
	No	20 (71.4)	32 (100)	19 (76)	17 (100)	
Qs3	Yes	24 (85.7)	21 (65.6)	21 (84)	12 (70.6)	0.213
	No	4 (14.3)	11 (34.4)	4 (16)	5 (29.4)	
Qs4	Yes	7 (25)	4 (12.5)	7 (28)	1 (5.9)	0.191
	No	21 (75)	28 (87.5)	18 (72)	16 (94.1)	
Qs5	Yes	19 (67.9)	2 (6.3)	19 (76)	1 (5.9)	**0.000**
	No	9 (32.1)	30 (93.8)	6 (24)	16 (94.1)	
Qs6	Yes	16 (57.1)	1 (3.1)	17 (68)	0 (0)	**0.000**
	No	12 (42.9)	31 (96.9)	8 (32)	17 (100)	

Data are presented as frequencies and percentages (in parentheses). A chi-square (χ^2^) test was performed, and a *p*-value ≤ 0.050 highlighted in bold was considered statistically significant. The questionnaire items are referred to by number, as described in the methodology section.

**Table 2:** Frequency of clinical and symptomatologic characteristics according to COVID-19 and Self-RPD status groups.

		COVID-19/SELF-RPD+ (*n* = 28)	COVID-19 (*n* = 32)	Periodontitis (*n* = 25)	Healthy Subjects (*n* = 17)	*p*
Age (years)		37.3 ± 16.6	36.2 ± 17.5	38.3 ± 15.3	41.4 ± 9.7	0.563
Gender	Female	15 (53.6)	18 (56.3)	12 (48)	10 (588	0.892
	Male	13 (46.4)	14 (43.8)	13 (529	7 (41.2)	
Smoking	Yes	6 (10.4)	6 (18.8))	11 (44)	3 (17.6)	0.114
	No	22 (78.6)	26 (81.3)	14 (56)	14 (82.4)	
Diseases	Yes	7 (25)	9 (28.1)	6 (24)	2 (12.8)	0.243
	No	21 (75)	23 (71.9)	19 (76)	15 (87.2)	
Drugs	Yes	17 (60.7)	10 (31.3)	12 (52)	5 (29.4)	0.075
	No	11 (39.3)	22 (68.8)	13 (48)	12 (70.6)	
Symptoms						
Fever	Yes	18 (64.3)	11 (34.4)	9 (36)	13 (76.5)	**0.006**
	No	10 (35.7)	21 (65.6)	16 (64)	4 (23.5)	
Dry cough	Yes	20 (71.4)	18 (56.3)	18 (72)	11 (64.7)	0.558
	No	8 (28.6)	14 (43.8)	7 (28)	6 (35.3)	
Nasal congestion	Yes	18 (64.3)	18 (56.3)	7 (28)	7 (41.2)	**0.045**
	No	10 (35.7)	14 (43.8)	18 (72)	10 (58.8)	
Tiredness	Yes	16 (57.1)	10 (31.3)	13 (52)	9 (52.9)	0.189
	No	12 (42.9)	22 (68.8)	12 (48)	8 (47.1)	
Cough with phlegm	Yes	7 (25)	16 (46.9)	4 (16)	2 (11.8)	**0.018**
	No	21 (75)	17 (53.1)	21 (84)	15 (88.2)	
Shortness of breath	Yes	4 (14.3)	3 (9.4)	2 (8)	1 (5.9)	0.808
	No	2 (85.7)	29 (90.6)	23 (92)	16 (94.1)	
Body aches	Yes	11 (39.3)	2 (6.3)	8 (32)	7 (41.2)	**0.011**
	No	17 (60.7)	30 (93.8)	17 (68)	10 (58.8)	
Headache	Yes	18 (64.3)	17 (53.1)	13 (52)	8 (47.1)	0.694
	No	10 (35.7)	15 (46.9)	12 (48)	9 (52.9)	
Chill	Yes	7 (25)	6 (18.8)	5 (20)	6 (35.3)	0.586
	No	21 (75)	26 (81.3)	20 (80)	11 (64.7)	
Muscle pain	Yes	9 (32.1)	9 (28.1)	8 (32)	9 (52.9)	0.360
	No	19 (67.9)	23 (79.1)	17(68)	8 (47.1)	
Joint Pain	Yes	5 (17.9)	10 (31.3)	8 (32)	5 (29.4)	0.613
	No	23 (82.1)	22 (68.8)	18 (68)	12 (70.6)	
Runny mucus	Yes	9 (32.1)	11 (34.4)	9 (36)	4 (23.2)	0.866
	No	19 (67.9)	21 (65.6)	16 (64)	13 (76.5)	
Burning throat	Yes	14 (50)	14 (43.8)	12 (48)	6 (35.3)	0.794
	No	14 (50)	18 (56.3)	13 (52)	11 (64.7)	
Flu	Yes	8 (28.6)	13 (40.6)	6 (24)	2 (11.8)	0.180
	No	20 (71.4)	19 (59.4)	19 (76)	15 (88.2)	
Conjunctivitis	Yes	6 (21.4)	9 (28.1)	6 (24)	2 (11.8)	0.651
	No	22 (78.6)	23 (71.9)	19 (76)	15 (88.2)	
Diarrhea	Yes	1 (3.6)	9 (28.1)	4 (16)	2 (11.8)	0.067
	No	27 (96.4)	23 (71.9)	21 (84)	15 (88.2)	
Vomit	Yes	1 (3.9)	6 (18.8)	3 (12)	2 (11.8)	0.350
	No	27 (96.4)	26 (81.3)	22 (88)	15 (88.2)	
Stomachache	Yes	3 (10.7)	2 (6.3)	6 (24)	1 (5.9)	0.160
	No	25 (89.3)	30 (93.8)	19 (76)	16 (94.1)	
Fast breathing	Yes	2 (7.1)	1 (3.1)	3 (12)	2 (11.8)	0.603
	No	26 (92.6)	31 (96.9)	22 (88)	15 (88.2)	
Convulsions	Yes	0 (0)	0 (0)	0 (0)	0 (0)	-
	No	28 (100)	32 (100)	25 (100)	11 (100)	
Olfactory disturbance	Yes	17 (60.7)	4 (12.5)	8 (32)	6 (35.3)	**0.001**
	No	11 (39.3)	28 (87.5)	17 (68)	11 (64.7)	
Gustatory disturbance	Yes	15 (53.6)	16 (50)	7 (28)	7 (41.2)	0.247
	No	13 (46.4)	16 (50)	18 (72)	10 (58.8)	

Data are presented as frequencies and percentages (in parentheses). A chi-square (χ^2^) test was performed for qualitative variables, and a Mann–Whitney U test was used to analyze age. A *p*-value ≤ 0.050 was considered statistically significant and highlighted in bold.

## Data Availability

The data supporting the findings of this study are available from the corresponding author upon reasonable request.
